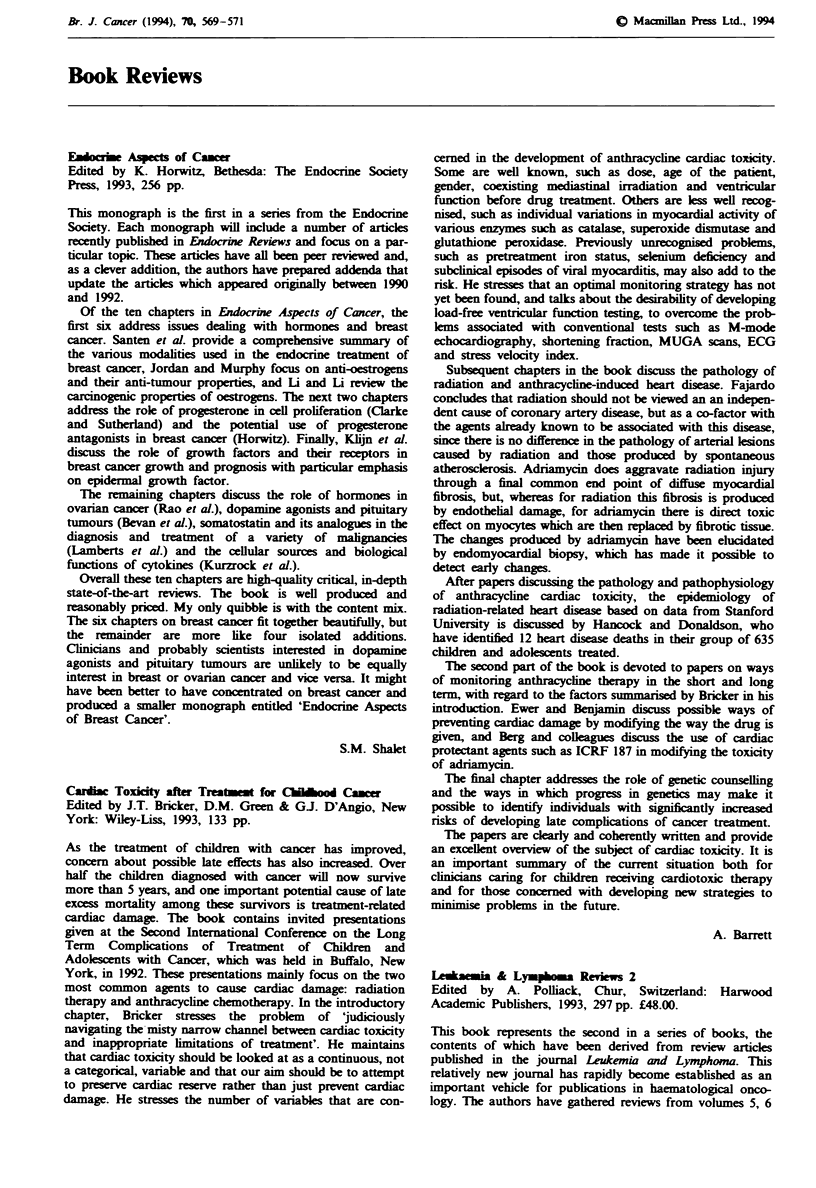# Endocrine aspects of cancer

**Published:** 1994-09

**Authors:** S.M. Shalet


 © Macmillan Press Ltd., 1994

### Book Reviews

**Endocrine Aspects of Cancer**
Edited by K. Horwitz, Bethesda: The Endocrine Society Press, 1993, 256 pp.

This monograph is the first in a series from the Endocrine Society. Each monograph will include a number of articles recently published in *Endocrine Reviews* and focus on a particular topic. These articles have all been peer reviewed and, as a clever addition, the authors have prepared addenda that update the articles which appeared originally between 1990 and 1992.

Of the ten chapters in *Endocrine Aspects of Cancer*, the first six address issues dealing with hormones and breast cancer. Santen *et al.* provide a comprehensive summary of the various modalities used in the endocrine treatment of breast cancer, Jordan and Murphy focus on anti-oestrogens and their anti-tumour properties, and Li and Li review the carcinogenic properties of oestrogens. The next two chapters address the role of progesterone in cell proliferation (Clarke and Sutherland) and the potential use of progesterone antagonists in breast cancer (Horwitz). Finally, Klijn *et al.* discuss the role of growth factors and their receptors in breast cancer growth and prognosis with particular emphasis on epidermal growth factor.

The remaining chapters discuss the role of hormones in ovarian cancer (Rao *et al.*), dopamine agonists and pituitary tumours (Bevan *et al.*), somatostatin and its analogues in the diagnosis and treatment of a variety of malignancies (Lamberts *et al.*) and the cellular sources and biological functions of cytokines (Kurzrock *et al.*).

Overall these ten chapters are high-quality critical, in-depth state-of-the-art reviews. The book is well produced and reasonably priced. My only quibble is with the content mix. The six chapters on breast cancer fit together beautifully, but the remainder are more like four isolated additions. Clinicians and probably scientists interested in dopamine agonists and pituitary tumours are unlikely to be equally interest in breast or ovarian cancer and vice versa. It might have been better to have concentrated on breast cancer and produced a smaller monograph entitled 'Endocrine Aspects of Breast Cancer'.

S.M. Shalet